# Energy Compensation Following a Supervised Exercise Intervention in Women Living With Overweight/Obesity Is Accompanied by an Early and Sustained Decrease in Non-structured Physical Activity

**DOI:** 10.3389/fphys.2019.01048

**Published:** 2019-08-22

**Authors:** Marie-Ève Riou, Simon Jomphe-Tremblay, Gilles Lamothe, Graham Stuart Finlayson, John Edward Blundell, Léa Décarie-Spain, Jean-Christian Gagnon, Éric Doucet

**Affiliations:** ^1^Behavioural and Metabolic Research Unit (BMRU), School of Human Kinetics, Faculty of Health Sciences, University of Ottawa, Ottawa, ON, Canada; ^2^Department of Mathematics and Statistics, Faculty of Science, University of Ottawa, Ottawa, ON, Canada; ^3^Biopsychology Group, Institute of Psychological Sciences, Faculty of Medicine and Health, University of Leeds, Leeds, United Kingdom

**Keywords:** exercise intensity, energy compensation, body composition, non-structured physical activity, doubly-labeled water, obesity

## Abstract

**Background/Objectives:**

Body composition (BC) does not always vary as a function of exercise induced energy expenditure (exercise EE – resting EE). Energy balance variables were measured to understand energy compensation (EC) in response to an exercise intervention performed at low (LOW) or moderate (MOD) intensity.

**Subjects/Methods:**

Twenty-one women with overweight/obesity (33 ± 5 kg/m^2^; 29 ± 10 yrs; 31 ± 4 ml O_2_/kg/min) were randomized to a 3-month LOW or MOD (40 or 60% of VȮ_2reserve_, respectively) matched to expend 1500 kcal/week (compliance = 97 ± 5%). Body energy stores (DXA), energy intake (EI) (food menu and food diaries), resting EE (indirect calorimetry), total EE (doubly-labeled water), time spent in different activities (accelerometers), appetite (visual analog scale), eating behavior traits and food reward (liking and wanting) were assessed at baseline, after weeks 1 and 2 and at the end of the 3-month exercise intervention.

**Results:**

EC based on BC changes (fat mass and fat-free mass) was 49 ± 79% and 161 ± 88% in LOW and MOD groups, respectively (*p* = 0.010). EI did not change significantly during the intervention. However, eating behavior traits and food reward had changed by the end of the 3-month supervised exercise. Non-structured physical activity (NSPA) decreased across the intervention (*p* < 0.002), independent of the intensity of the exercise training.

**Conclusion:**

Women with overweight/obesity training at LOW presented lower EC for a given energy cost of exercise. Our results strongly suggest that NSPA plays a major role in mediating the effects of exercise on energy balance and ultimately on changes in BC.

**Clinical Trial Registration:**

www.ClinicalTrials.gov, identifier ISRCTN31641049.

## Background/Objectives

The impact of exercise EE on weight loss is often less than expected (Miller et al., 1997). The limited dose of prescribed exercise (Thomas et al., 2012), the lack of compliance, the reduction of resting EE, the increase of EI and/or the decrease in NSPA following exercise are among the reasons that have been proposed to explain this observation (Thomas et al., 2012; Caudwell et al., 2013). We recently reported results from a systematic review, which showed an overall EC from exercise interventions of ∼ 20%, with EC being closer to 100% for longer interventions (Riou et al., 2015a).

Evidence suggests little EI compensation after acute exercise bouts [(King et al., 1994, 1997; Imbeault et al., 1997), and there are several reviews on this topic (King, 1999; Martins et al., 2008; King et al., 2012)]. However, following 14 days, EI compensation seems to hover around 30% (Stubbs et al., 2002a; Blundell et al., 2003; Whybrow et al., 2008), a phenomenon potentially influenced by the sex of subjects (Andersson et al., 1991; Meijer et al., 1991; King et al., 1996; Todd Alan Hagobian, 2012), eating behavior traits and hedonic processes (Lluch et al., 1998, 2000; Finlayson et al., 2009) as well as adiposity levels (Woo et al., 1982a, b; Kissileff et al., 1990; Westerterp-Plantenga et al., 1997; George and Morganstein, 2003; Hagobian et al., 2009) [for reviews see Hill et al. (1995) and Martins et al. (2008)]. EI following exercise is also subject to large inter-individual variability (Hopkins et al., 2013). Finlayson et al. (2009) showed that lean women who presented a higher EI following an acute bout of exercise also displayed a higher preference for high-fat sweet food and generally found foods more palatable. Similarly, it has been reported that compensators (individuals with a weight loss smaller than predicted) present an increase in EI with a greater intake from fat and increased hunger (King et al., 2008).

Some literature supports an increase in sedentary activities after exercise, which could ultimately result in no change in total EE, and may in part explain why the effects of exercise on BW are dampened (Goran and Poehlman, 1992; Morio et al., 1998; Meijer et al., 1999, 2000; Colley et al., 2009). However, NSPA (transportation, work-related movement and/or activity of daily living) (Levine, 2004; Levine et al., 2006; Colley et al., 2009) has also been found to remain unchanged (Blaak et al., 1992; McLaughlin et al., 2006; Rangan et al., 2011; Martin et al., 2019; Myers et al., 2019), or to increase (Meijer et al., 1991; Westerterp et al., 1992; Hollowell et al., 2009) in response to exercise interventions.

Exercise intensity could also influence changes in body energy stores. Results from lean and overweight individuals suggest that BW decreased to a greater extent in response to lower intensity (LOW) than from higher intensity (HI) exercise intervention (Grediagin et al., 1995; Mougios et al., 2006). In contrast, no changes in BW and BC were noted following exercise training performed at either LOW or HI in individuals living with obesity (Hinkleman and Nieman, 1993; van Aggel-Leijssen et al., 2001, 2002). Collectively, these observations suggest that multiple factors may hinder exercise-induced weight loss (Myers et al., 2014). There is a need to more clearly understand the impact of exercise on factors that could ultimately influence EI and total EE.

The objective of this study was to provide a comprehensive investigation of the impact of an equicaloric (1500 kcal/week) 3-month exercise intervention performed at LOW or moderate (MOD) intensity (40% VȮ_2reserve_ or 60% VȮ_2reserve_, respectively) on EC. It was hypothesized that women training at MOD would have higher EC across the intervention due to greater EI and to a decrease in NSPA, when compared to women training at LOW.

## Subjects and Methods

### Participants

**FIGURE 1:**
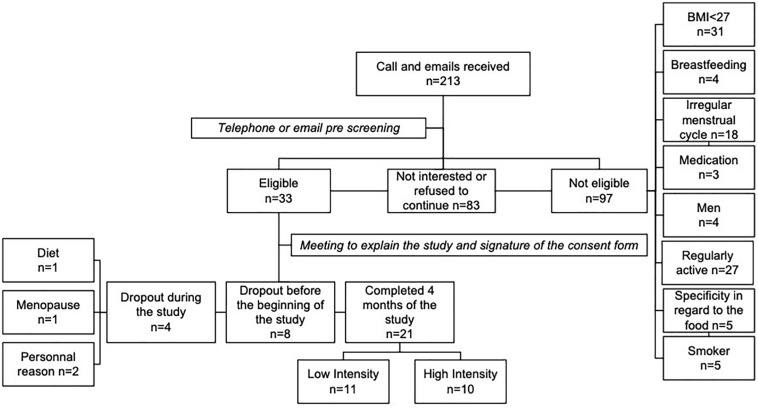
Recruitment and sample size of the study.

Twenty-five premenopausal women with overweight/obesity aged between 19 and 51 years were recruited to participate in a 3-month supervised exercise intervention. The inclusion criteria were as follows: (1) regular menstrual cycle; (2) non-smoker; (3) body mass index (BMI) higher than 27 kg/m^2^; (4) reported weight stability (±2 kg) for ≥2 months prior to enrolment in the study; and (5) physically inactive (practice of exercise <150 min/week). All participants were healthy with no orthopedic limitations, were not taking any medications that had the potential to impact study outcomes and had no history of alcohol (<2 drinks/day) or drug abuse. As presented in Figure 1, 4 women withdrew from the study due to personal reasons (*n* = 2), dieting (*n* = 1) and irregular menstrual cycle (*n* = 1). As such, twenty-one participants were included in the analyses. Participants were not aware of the true purpose of this study. They were told that the objective was to determine how the program will increase their physical fitness. This study was conducted according to the guidelines laid down in the Declaration of Helsinki and the University of Ottawa ethics committees approved all procedures involving human subjects. Written informed consent was obtained from all subjects.

### Experimental Procedures

**FIGURE 2:**
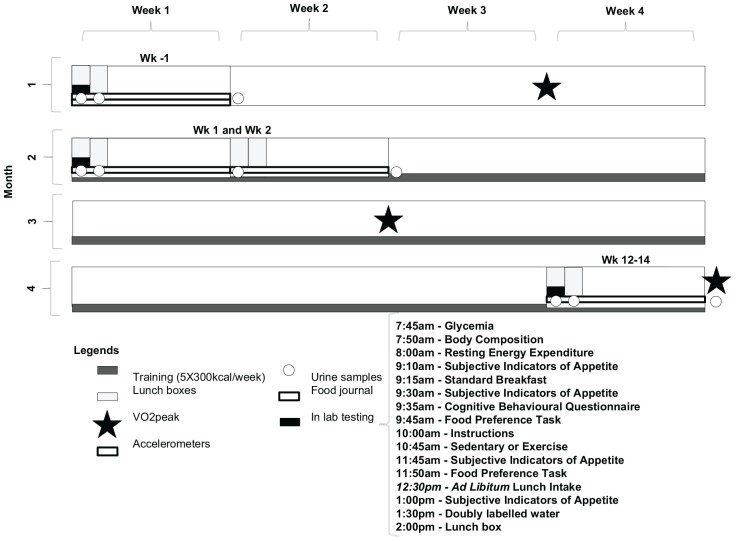
Study design.

This study had three data collection phases: 7-day phase at baseline, which was completed 1 month before the exercise intervention (wk -4); a 14-day phase at the onset of the exercise intervention (wk 1, wk 2); and a 7-day phase at the end of the 3-month exercise intervention (wk 12) (Figure 2). Women were tested between days 1–9 of the follicular phase of the menstrual cycle at all sampling points. Due to the different length of the menstrual cycle, the testing at the end of the exercise intervention varied between wk 12 and wk 14. This is also the reason for which 1 month elapsed between baseline testing and the onset of the exercise intervention.

### In Laboratory Morning Testing (wk -4, wk 1, wk 12–14)

On the first day of each phase, BW and BC (7:50 am) and resting EE (8:00–9:00 am) were measured. Anthropometric measurements are described elsewhere (McNeil et al., 2012). BC was measured with a DXA system (GE-LUNAR Prodigy module; GE Medical Systems, Madison, WI, United States). Coefficients of variation and correlation for %BF measured in twelve subjects tested in our laboratory were 1.8% and *r* = 0.99, respectively.

Resting energy expenditure (REE) was assessed by indirect calorimetry using the ventilated hood technique and the Vmax Encore 29 N metabolic cart (SensorMedics Corporation, Yorba Linda, CA, United States). The metabolic carts were calibrated against 95% O2/5% CO2 reference gas at the beginning of each day. Concentrations of CO_2_ and O_2_ were measured during 30 min (5 min of acclimatization and the average of the following 6th–25th min were included in the analysis) and Weir’s equation (Weir, 1949) was used to determine the caloric equivalents of oxygen consumption. Measurements were taken between 7:00 and 9:30 am after 12-h fasting period, while also abstaining from exercise for at least 24 h. This measurement was always done in the same room, which was kept dark and at an ambient temperature of ∼21° Celsius. After a 20–30-min resting period in the supine position, 30 min of measurements were conducted, whereas only the last 20 min were used in the calculation of REE. Ethanol burning tests are routinely performed to control the quality of measurements, as previously described (Cooper et al., 2009; Blond et al., 2011). Our most recent analyses demonstrated −5% and 4.76% difference in CO2 and O2 measures, respectively.

Appetite (i.e., desire to eat, hunger, fullness and prospective consumption) was measured using visual analog scales before (9:10 am) and after (9:30 am) breakfast (Hill et al., 1984; Marsh-Richard et al., 2009).

Breakfast (9:15 am) included *ad libitum* whole wheat bread, strawberry jam, peanut butter, cheddar cheese and orange juice. The amount (kcal) and the food selected at wk -4 were used for the following two sampling points (wk 1 and week 12–14). As such, breakfast was standardized for each individual but differed from one individual to the next.

Eating behavior traits (9:35 am) were measured with the Three-Factor Eating Questionnaire (Stunkard and Messick, 1985). The LFPQ, a forced choice computer task (9:45 am) was used to evaluate the implicit hedonic wanting (speed and frequency of choice) and the explicit hedonic liking (subjective VAS rating) for different visual food cues, which varied in both fat and sugar content (Finlayson et al., 2007).

Instructions for urine sample collection (DLW) and for food diaries were then provided to participant (10:00 am). At baseline (wk -4), the hour that followed (10:45–11:45 am) comprised of a sedentary session (e.g., writing, reading, studying, etc.), while exercise was performed on the first day of wk 1 and wk 12–14.

### In Laboratory Afternoon Testing (wk -4, wk 1, wk 12–14)

Appetite was measured before and after lunch (11:45 am and 1:00 pm), while the LFPQ was performed before lunch (11:50 am).

*Ad libitum* intake was measured with a food menu that was presented to participants (12:00 pm), so that they could choose the foods and beverages that they wanted to consume (McNeil et al., 2012). After lunch, participants were asked to choose (1:15 pm) foods and beverages offered *ad libitum* from the same food menu for the rest of the day as well as for the following day (1.5 days).

### Free-Living Environment (wk -4, wk 1, wk 12–14)

Participants received the foods they had selected (2:00 pm) and were instructed to bring back all leftovers and wrappers. Participants were also instructed as to how to complete the 7-day food diaries.

Participants were fitted with two triaxial accelerometers around the arm and thigh (SenseWear Pro 3 Armbands^©^, HealthWear Bodymedia, Pittsburgh, PA, United States). Data from the accelerometers were used to measure the time spent in different activities (i.e., time spent walking, standing, sitting and lying down). The following method was developed and validated in our laboratory (Riou et al., 2015b). Briefly, the data (acceleration axes and step counts) were obtained from accelerometers worn by participants. The INNERVIEW software (version 4.02; Bodymedia, Pittsburgh, PA) was used to extract the data obtained from accelerometers. Training data were exported in two Comma-Separated Values (CSV) files: one file for the accelerometer worn on the arm and one for the accelerometer worn on the thigh. Activity Recognition software was used to combine and to synchronize these two training data files, which produced a single file containing a sequence of training data samples. Then, the SVM model was used to classify each data sample as an activity (e.g., classified as variants of lying down, standing and sitting). The total time spent performing each activity was determined as the product of the sampling rate (5 s) and the number of occurrences of the different activities.

Urine samples were collected on the following morning as well as on the morning of day eight in order to assess total EE with DLW (Schoeller and van Santen, 1982). As previously described by St-Onge et al. (2007), we used differential loss of the 2H and 18O isotopes of water to integrate carbon dioxide production over time in free-living subjects. Following the administration of deuterated water, 2H is lost from the body water at an exponential rate. When 18O-water is administered, the 18O is also lost with body water turnover (as per the 2H isotope) and with each molecule of carbon dioxide produced because carbonic anhydrase in the body rapidly facilitates the equilibrium exchange of water and carbon dioxide/carbonic acid oxygen (Speakman et al., 1993; Racette et al., 1994). The difference between the rates of disappearance of 2H and 18O corresponds to the total carbon dioxide production over that period. The rates of disappearance are determined from urine samples taken at the start and at the end of the measurement period. Based on results from food journals administered at baseline and after the intervention, the food quotient was calculated and used to establish oxygen consumption and to consequently obtain a value for daily EE. Daily energy spent performing physical activity was calculated by subtracting REE from daily EE, while assuming that the thermic effect of food (TEF) remained stable (at 10% of daily EE). The DLW measurement periods generated 5 urine samples per subject: a pre-dose sample, 2 samples obtained after the 2H218O dose has initially equilibrated in the body (post-dose samples 1 and 2), and 2 at the end of the collection period (post-dose samples 3 and 4). Post-dose samples 1 and 2 were collected on day 1, 16–24 h after the dose of DLW on day 0. There was a minimum of 30 min and a maximum of 4 h between post-dose samples 1 and 2 as well as between post-dose samples 3 and 4. All samples were measured in triplicate for 18O-water and for 2H-water. An Isoprime Stable Isotope Ratio Mass Spectrometer connected to a Multiflow-Bio module for Isoprime and a Gilson 222XL Autosampler (GV Instruments, Manchester, United Kingdom) was used. Data processing was performed with MassLynx 3.6 software (Waters Corp, Milford, MA). Stability tests were performed each day before testing, which yielded an SD of 0.026% for deuterium and 0.004% for 18O. Known reference materials—Vienna-Standard Mean Ocean Water (V-SMOW), Greenland Ice Sheet Program (GISP), Standard Light Antarctic Precipitation (SLAP), and International Atomic Energy Agency standards (IAEA-304A and IAEA-304B)—were used for calibration and data normalization. Isotope ratio analysis results were reported as delta relative to a reference gas.

### Free-Living Environment (wk 2)

For the first 2 days of wk 2, participants were asked to self-select foods from the menu and received two lunch boxes containing foods selected. They were instructed to wear the armbands and to fill out the food diary diaries for a second week. Urine samples were collected on the last day of this second week in order to assess total EE with the DLW method.

### Cardiorespiratory Fitness (VȮ_2peak_)

A progressive exercise stress test was performed to measure participants’ peak maximal oxygen consumption (VȮ_2peak_) at wk -4, 6 wk after the onset of exercise and at wk 12–14. Breath-by-breath samples of expired air were collected with a mouthpiece, and measurements of VO2 and respiratory exchange ratio were automatically collected using a Vmax 229 series metabolic cart (SensorMedics Corporation, Yorba Linda, CA, United States). Each test lasted between 8 and 12 min and we followed the ramp medium protocol test on a treadmill until exhaustion. The ramp medium protocol test on a treadmill was used until exhaustion. The test was terminated when at least 2 of the following criteria were reached (Whaley et al., 2006): (1) predicted maximal HR reached, (2) respiratory quotient >1.1, (3) oxygen consumption remained stable or decreased with an increase in workload, or (4) rate of Borg-type scale reached ≥19. The VȮ_2reserve_ = [(VȮ_2peak_–resting O_2_ ml min^–1^) × Intensity (40% (LOW) or 60% (MOD)) + resting O_2_ ml min^–1^] was used to prescribe the corresponding target HR for the exercise intervention (HR-VȮ_2reserve_ regressions).

### Exercise Intervention

The 21 participants were randomly assigned to one of the two intensity exercise interventions. Eleven participants trained at LOW (40% of the VȮ_2reserve_) while 10 participants trained at MOD (60% of the VȮ_2reserve_). The intensity of each training session was precisely monitored with a HR monitor (Polar RS300) to achieve 300 kcal per exercise session (as described above), which is consistent with the recommendations given by the ACSM for weight loss and health benefits (Donnelly et al., 2009). Participants took part in 3 supervised training sessions in the laboratory and performed 2 by themselves. Most trained on a treadmill and while 2 participants (MOD) used a cycle-ergometer. HR was recorded (Polar RS300) for all sessions for the entire intervention. The target HR was set to ± 10 beats of the prescribed HR. When HR deviated outside of this range, treadmill speed and/or incline was adjusted accordingly (resistance and/or cadence for the cycle ergometer). HR data was downloaded weekly to track compliance and to determine average HR/session. The average HR/session was used to determine the VO_2_ value derived from the HR-VO_2_ relationship collected during the VO_2max_. The total net energy cost of exercise, which includes every training session, and is calculated as “exercise EE – resting EE,” was then calculated using the Weir formula (Weir, 1949). To account for training adaptation, VȮ_2peak_ performed at wk -4 was used to adjust the intensity of the exercise intervention for the first 6 weeks while the VȮ_2peak_ performed at 6 wk after the onset of exercise was used to adjust for the remainder of the study. Due to the variable length of the menstrual cycle and since all testing were performed during the follicular phase, women trained on average for 14 ± 2 weeks.

### Energy Compensation

EC was calculated from the total exercise EE (kcal) and changes in FM and FFM (kg converted to kcal) over the course of the intervention (wk 12–14 – wk 1) (Riou et al., 2015a). Due to technical problems with measurements obtained from the DXA at wk 1 (*n* = 1), data were taken from wk -4. Changes in body energy were calculated as follows: a gain/loss of 1 kg of FM corresponds to 9500 kcal, while it corresponds to 1200 kcal for FFM (Hall, 2008). A decrease in FM and/or FFM was entered as a negative value, whereas a gain in these variables was entered as a positive value. EC (%) was thus calculated as follows:

$$\frac{100}{\mathrm{Energy}\,\mathrm{Expenditure}\,\mathrm{from}\,\mathrm{Exercise}\,\left(\text{kcal}\right)}\times \left[\left(\Delta \,\text{FM}\,\left(\text{kg}\right)\times 9500\,\mathrm{kcal}\right)+\left(\Delta \,\text{FFM}\,\left(\text{kg}\right)\times 1200\,\mathrm{kcal}\right)\right]+100$$

A compensation of 0% represents BC variations perfectly aligned with exercise EE. In contrast, a compensation of 100% indicates that BC remained the same despite exercise EE. Finally, when compensation is negative body energy stores are reduced beyond what is expected from the amount of energy spent during exercise.

### Statistical Analysis

**FIGURE 3:**
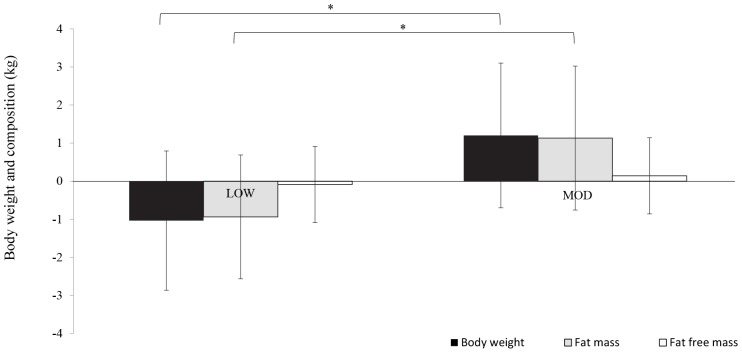
Changes in body weight and composition during the exercise intervention performed at LOW (*n* = 11) and MOD (*n* = 8). Variables were expressed as change scores. LOW group: Body weight = –1.0 ± 1.8; Fat mass = –0.9 ± 1.6; Fat-free mass = –0.1 ± 1.7. MOD group: Body weight = 1.2 ± 1.9; FM = 1.1 ± 1.9; Fat-free mass = 0.1 ± 2.7. ^∗^, Indicates a significant main effect of group.

**FIGURE 4:**
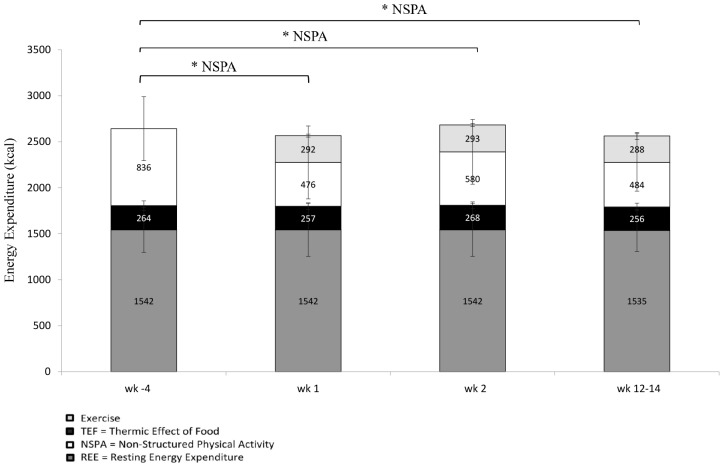
Components of total energy expenditure measured with indirect calorimetry and doubly-labeled water (REE, NSPA, exercise induced EE) [LOW (*n* = 11) and MOD (*n* = 10)]. TEF was not measured and fixed at 10% of total daily EE. ^∗^NSPA, indicates a significant main effect of phase (*p* = 0.002, cohen’s *f*^2^ = 0.299).

Descriptive variables are presented as means ± SD. Normality was assessed with a Shapiro-Wilk test with variables and residuals. Q-Q plots were also generated to investigate the normality of the distribution. Differences between LOW and MOD for baseline characteristics (Table 1), exercise intervention (Table 2) and EC were assessed using an independent sample t-test. In the case of violations of normality, a Mann-Whitney *U*-Test was performed. A two-way mixed model ANOVA with a within-subject factor of “phase” (wk 1 and wk 12–14) and a between-subjects factor of “intensity” [2 levels: LOW (± 40% of VȮ_2reserve_ and MOD: ± 60% of VȮ_2reserve_)] was used to analyze the dependent variables of BC (Figure 3). A two-way mixed model ANOVA with a within-subject factor of “phase” (wk -1, wk 1, wk 2, and wk 12–14) and a between-subject factor of “intensity” [2 levels: LOW (± 40% of VȮ_2reserve_ and MOD: ± 60% of VȮ_2reserve_)] was used to analyze the dependent variables of EI and macronutrients, subjective indicators of appetite, eating behavior traits and food reward as well as EE (Figure 4). When significant differences were found, *post hoc* test analyses were performed with paired *t*-tests and Bonferroni corrections were applied for multiple comparisons.

**TABLE 1:** Participants’ characteristics at baseline.

	**LOW**	**MOD**	***P*-values**	**Cohen’s d**
n	11	10		
Age (y)	27 ± 9	31 ± 11	0.28^1^	0.40
***Body composition***				
Body weight (kg)	88.1 ± 12.0	94.9 ± 21.1	0.37	0.40
BMI (kg⋅m-2)	32.3 ± 3.8	35.1 ± 6.2	0.22	0.55
Fat mass (kg)	41.9 ± 8.2	45.0 ± 14.2	0.55	0.27
Percent fat mass (%)	47.9 ± 3.4	47.1 ± 5.3	0.71	0.18
Fat free mass (kg)	45.0 ± 4.3	48.5 ± 6.7	0.17	0.63
***Energy Intake (kcal)***				
n	11	8		
Breakfast	648 ± 166	746 ± 256	0.33	0.47
Lunch	772 ± 322	676 ± 330	0.54	0.30
Day 1	2620 ± 780	2842 ± 959	0.59	0.26
Day 2	2418 ± 659	2443 ± 1050	0.95	0.03
Day 3–7	2048 ± 378	2290 ± 718	0.40	0.44
Day 1–7	2182 ± 410	2391 ± 714	0.48	0.38
***Energy Expenditure (kcal/day)***				
n	11	10		
REE	1469 ± 185	1623 ± 285	0.16	0.65
NSPA	701 ± 272	985 ± 371	0.06	0.88
TEE	2411 ± 289	2898 ± 596	0.04^2^	1.06
***Eating behaviors traits***				
n	11	8		
Dietary restraint	8.6 ± 3.2	9.4 ± 2.7	0.61	0.27
Flexible dietary restraint	2.0 ± 0.8	1.8 ± 1.0	0.48^1^	0.23
Rigid dietary restraint	1.6 ± 1.4	2.5 ± 0.5	0.67^1^	0.80
Disinhibition	8.5 ± 2.9	8.5 ± 1.5	0.97	0.02
Hunger	7.0 ± 2.6	7.5 ± 2.9	0.70	0.18

*Values are mean ± SD. LOW, lower intensity; MOD, moderate intensity; BMI, body mass index; REE, resting EE; NSPA, non-structured physical activity; TEE, total EE ^1^A Mann Whitney *U*-Test was performed due to the non-normal distribution of the variables. ^2^When Total EE was expressed as a function of BW, no significant difference was noted between both groups (LOW = 30.12 ± 3.97 (*n* = 11) vs. MOD = 28.08 ± 4.08 (*n* = 10); *p* = 0.26, cohen’s *d* = 0.51).*

**TABLE 2:** Characteristics of the exercise intervention.

	**LOW**	**MOD**	***P*-values**	**Cohen’s d**
*n*	11	10		
Characteristics of the exercise EE intervention				
Weeks of training	14 ± 1	15 ± 2	0.24^1^	0.64
Estimated training sessions	68 ± 6	75 ± 12	0.16	0.75
Training sessions completed	68 ± 6	71 ± 12	0.58	0.32
Compliance (%)	100 ± 2	95 ± 6	0.03^1^	1.14
Exercise EE per session (kcal)	352 ± 20	343 ± 27	0.40	0.38
Exercise above REE per session (kcal)	290 ± 18	292 ± 25	0.87	0.09
Total exercise EE for the intervention (kcal)	23902 ± 2584	24015 ± 4173	0.94	0.03
Time spent exercising (min)/session	62 ± 6	46 ± 6	<0.000	2.67
VȮ_2peak_ (ml/kg/min)	32 ± 4	30 ± 4	0.38	0.5
Mean HR during exercise (bpm)	117 ± 10	134 ± 15	0.006	1.35

*Values are mean ± SD. NS, not significant; LOW, lower intensity; MOD, moderate intensity; EE, energy expenditure; REE, Resting EE; VȮ_2peak_, participants’ peak maximal oxygen consumption; HR, heart rate. ^1^A Mann Whitney *U*-Test was performed due to the non-normal distribution of the variables.*

Prior to the analyses, imputations (Elliott and Hawthorne, 2005) (i.e., mean of LOW and mean MOD group at a specific time) were performed for 6 samples: 3 for the DLW (i.e., sampling limited in isotopes, problem with the time of urine collected), 2 for exercise EE and 1 for resting EE. NSPA was obtained by subtracting resting EE, TEF (estimated at 10% of total EE) and exercise EE from total EE. Data obtained from accelerometers were used to measure the time spent performing different activities (i.e., time spent walking, standing, sitting and lying down). To be included into the analyses, accelerometers had to be worn for more than 65% of the day (936 min over 1440 min). For these analyses, 8 and 6 participants were part of the LOW and MOD groups. Data obtained across the intervention (wk -4, wk 1, wk 2 and wk 12–14) were presented as the mean of time spent in different activities on the day where a training was or was not performed.

## Results

### Baseline Participants’ Characteristics and BC Changes

Baseline characteristics are shown in Table 1, while exercise data are presented in Table 2. No main effects, no group effects and no interactions were noted for maximal oxygen consumption (data not shown). The analyses revealed a combined EC of 51 ± 173% and no significant difference between LOW and MOD participants, respectively [49 ± 79% (*n* = 11) and 53 ± 244% (*n* = 10) (cohen’s *d* = 0.02)]. However, further analyses, based on median comparison (*p* = 0.086) and boxplot, demonstrated extreme variables. When removed, a significant difference was noted for EC between groups [LOW: 49 ± 79% (*n* = 11) and MOD: 161 ± 88% (*n* = 8), *p* = 0.010, cohen’s *d* = 1.35]. Analyses suggested significant group interactions for body FM (*p* = 0.020, cohen’s *f*^2^ = 0.278) and BW (*p* = 0.019, cohen’s *f*^2^ = 0.283) (Figure 3). Since the extreme negative compensation of the two outliers was almost certainly explained by the addition of a caloric restriction, these participants were not included in the analyses of EI, subjective indicators of appetite, eating behavior traits and food reward (LOW, *n* = 11 and MOD, *n* = 8). However, all participants, including the outliers for EC, were included in the analyses of EE variables (LOW, *n* = 11 and MOD, *n* = 10).

### Energy Intake, Macronutrient and Subjective Indicators of Appetite

No significant differences were noted between women training at LOW and MOD for EI and macronutrient at breakfast. No main effect of phase, group, or group by phase interactions were noted for EI and macronutrient at lunch, on day 1 and 2 or for the mean value over 7 days (i.e., 2 days of food menu and 5 days of food diary).

After the exercise session, women training at MOD had a higher degree of fullness and had lower mean prospective food consumption when compared to women training at LOW (data not shown). After lunch, a trend (*p* = 0.083, cohen’s *f*^2^ = 0.162) was notice for mean prospective food consumption during the intervention (i.e., 4 ± 5 mm, 6 ± 7 mm, 13 ± 22 mm, respectively). Appreciation of the foods consumed from the food menu after breakfast and after lunch was similar across the intervention with no significant effect of group, or group by phases interaction (82 ± 18 mm and 89 ± 11 mm, respectively).

### Eating Behavior Traits (TFEQ) and Food Reward

Hunger and external hunger significantly increased (*p* = 0.002, cohen’s *f*^2^ = 0.315 and *p* = 0.000, cohen’s *f*^2^ = 0.436, respectively for both traits) from wk -4 to wk 1 (*p* = 0.032 and *p* = 0.000, respectively) and from wk -4 to wk 12 (*p* = 0.004 and *p* = 0.002, respectively). No significant difference was noticed for other eating behavior traits. Implicit wanting for fat decreased from wk 1 to wk 12–14 (*p* = 0.034, cohen’s *f*^2^ = 0.237). Implicit wanting for fat (*p* = 0.000, cohen’s *f*^2^ = 0521) and explicit liking for fat (*p* = 0.025, cohen’s *f*^2^ = 0.261) significantly decreased after the exercise session when compared to before. Women in the LOW group demonstrated higher implicit wanting for fat when compared to women training in the MOD group (*p* = 0.054, cohen’s *f*^2^ = 0.202). Following the exercise session, a trend was noticed for implicit wanting for savory foods increased (vs. sweet) (*p* = 0.063, cohen’s *f*^2^ = 0.189). An interaction between phase and time (*p* = 0.002, cohen’s *f*^2^ = 0.443) was shown for explicit liking for savory foods. When compared to before the exercise session, explicit liking for savory foods increased after the exercise session at wk 1, while it decreased at wk 12–14.

### Energy Expenditure and Time Spent Performing Activities

Components of total EE (resting EE, NSPA, exercise induced EE) are presented in Figure 4. No significant difference was noted for resting EE and total EE across the intervention. A trend (*p* = 0.055, cohen’s *f*^2^ = 0.181) between LOW and MOD group was found for total EE, where MOD participants tended to have greater total EE when compared to LOW participants. No interaction was observed. Significant main effects of phase (*p* = 0.002, cohen’s *f*^2^ = 0.299) (Figure 4) were noted for NSPA. *Post hoc* comparisons revealed significant decreases between wk -4 and wk 1 (*p* = 0.006), wk -4 and wk 2 (*p* = 0.036) and wk -4 and wk 12–14 (*p* < 0.000).

Time spent walking significantly increased (*p* < 0.000, cohen’s *f*^2^ = 0.446) from wk -4 to wk 1 (*p* < 0.003), wk -4 to wk 2 (*p* = 0.003), wk 12–14 (*p* = 0.021). LOW participants spent significantly more time walking then MOD participants (*p* = 0.046, cohen’s *f*^2^ = 0.292). An interaction was noted for the time spent lying down (*p* = 0.044, cohen’s *f*^2^ = 0.199). Across the intervention, MOD participants significantly increased their time spent lying down while LOW participants decreased it. No other differences were observed for posture allocation (i.e., sitting, standing, running).

## Discussion

This study measured EC in women living with overweight/obesity training at either LOW or MOD over the course of a 3-month (12–14 wks) intervention. Our data suggest that on average, exercise EE was almost entirely compensated (96%), an effect that was much greater in women training at MOD (161%) when compared to women training at LOW (49%). DLW samples were taken during 4 of the 12 wks of the protocol, which provide comprehensive coverage of the effects of exercise on EE. During the same period, EI was monitored with lunch boxes and 7-day dietary records, while body energy changes were measured with DXA. The close monitoring of EE from the exercise revealed a high level of compliance to the exercise sessions over the course of the study (∼97%). While EI measures did not change across the intervention, our results emphasized that total EE, measured with DLW, did not increase during the exercise intervention in either of the groups. What is more, this effect was noted as early as the onset of the exercise intervention (wk 1). As well, NSPA decreased across the intervention, independently of the intervention group.

Our study suggest that total EE remained stable across the intervention. More specifically, NSPA decreased early and remained lower during the entire exercise intervention. Results from previous studies have shown a decrease in NSPA in elderly subjects (Goran and Poehlman, 1992) in response to an exercise intervention as well as in overweight boys (Paravidino et al., 2016) following an acute bout of exercise. On the other hand, others have found that individuals living with overweight and obesity do not decrease the time spent performing sedentary activities (Willis et al., 2014). Our results also demonstrate that the amount of time spent lying down increased across the intervention for MOD while it decreased for LOW. In addition, the time spent walking increases in both group and the analyses suggested that the amount was significantly higher in LOW when compared to MOD. Nevertheless, our characterization of posture allocation did not align with the changes in NSPA measured with DLW. Even if our classification model was found to be accurate and reproducible (Riou et al., 2015b), it could be speculated that our wear time cut-off (65%) could have been too low to fully capture differences in posture allocation time.

Our results are in agreement with literature as far as showing that EI does not increase following acute exercise or over a 2-day period when compared to baseline values [(King et al., 1994, 1997; Imbeault et al., 1997); for reviews, see King (1999), Martins et al. (2008), King et al. (2012)]. However, for long-term exercise performed at a HI, we could have anticipated an increase in EI due to the documented high level of EI compensation under similar conditions (Stubbs et al., 2002b; Pomerleau et al., 2004; Whybrow et al., 2008; Martin et al., 2019; Myers et al., 2019). However, no significant effect was noted for time or group during the intervention. Similar results were also obtained in men and women living with overweight/obesity expending 1500 kcal/week or 3000 kcal/week during a 12 week intervention (Flack et al., 2018) and in an intervention designed to expend 300 kcal and 600 kcal for 13 wks in men with overweight (Rosenkilde et al., 2012).

When considering other important contributors to EI variability (King, 1999; King et al., 2012), such as hedonic responses (i.e., subjective indicators of appetite and eating behaviors traits), results are in line with the relatively stable EI observed during the intervention. Our analyses suggest that implicit hedonic wanting for fat and explicit hedonic liking for fat decreased after the exercise session, a result that persisted over time for implicit hedonic wanting for fat. Interestingly, comparable results were found in non-compensators after an acute exercise (Finlayson et al., 2009). The decrease for explicit liking for sweet before the exercise session, when compared to the increase after the exercise session between wk 1 and wk 12–14 is in accordance with an increased preference for carbohydrate following acute exercise (Westerterp-Plantenga et al., 1997). Additionally, subjective indicators of appetite revealed that women training at HI presented a higher acute degree of fullness and had lower mean prospective food consumption after the exercise session when compared to women training at LOW (Panissa et al., 2016). Finally, our results suggest that hunger and external hunger as assessed with the TFEQ, increased across the intervention. However, we previously demonstrated that this result was associated with EI among overweight women performing a lower level of physical activity but not related to EI for women with a higher physical activity level (Riou et al., 2011).

Because our results, EI measurements did not change significantly over the course of the study. It would seem logical to conclude that the compensation from exercise-induced EE was not mediated by variations EI. This perception is supported by the fact that we assessed dietary intake over 7 days at four different time points during the entire study with a combination of objective food intake (McNeil et al., 2012) and self-report. However, it is important to remember that any changes in EE, including those in NSPA, can only impact body energy stores if they create a prolonged energy imbalance. Along these lines, our results show that from baseline to the end of the intervention mean total EE for LOW essentially remained the same (∼+5 kcal/day or +490 kcal for the intervention), whereas a non-significant decrease in mean total EE was noted for MOD (∼−173 kcal/day or −18165 kcal for the intervention). Mean calculated body energy changes for the intervention were −8502 and +10379 kcal for LOW and MOD, respectively. This means that changes in EE cannot by themselves explain the changes in body energy stores that were measured, even if NSPA was significantly reduced for both groups. If we assume that our calculations are accurate, this means that EI was decreased by approximately ∼8012 kcal or 82 kcal/day (−8502+490 kcal) for LOW and by ∼7786 kcal or 74 kcal/day (10379–18165 kcal) for MOD during the entire trial. Arguably, such subtle daily differences are extremely challenging to reveal considering the sensitivity of the tools used to measure free-living EI in this study and elsewhere. These numbers also highlight the inherent challenges related to the precise evaluation of EI in the context of comprehensive energy balance studies (Schoeller et al., 2013).

Since the BMI at inclusion was >27, these conclusions cannot be extended to the general population. The high quantity and quality of the variables measured lead to the inclusion of a relatively smaller number of participants. Consequently, the results have a lower statistical power. Nevertheless, the low effect size noted for important primary outcomes suggests that a higher number of participants would have very likely led to similar results. It has been suggested that greater weight loss could have been obtained with a larger dose of exercise (Jakicic et al., 2003). However, it should be emphasized that three out of five sessions were performed and monitored in the laboratory and that two other sessions were carefully scrutinized with a HR monitor. Exercise compliance was 97% for this study. Since measurement with indirect calorimetry would have been too laborious (21 participants × 5 trainings per week× 3 months), the estimation of exercise EE is a limitation of this study. As well, exercise-induced increases in RMR (EPOC effect) was not considered in this study. Nevertheless, as discussed in the review from LaForgia et al. (2006), a sufficient stimulus (for example, a 50 min at 70% of VO2max) would be enough to create an additional deficit of 6–15% of the net total oxygen cost of the exercise. Considering these values, the EPOC from our study would be between 17 and 44 kcal, and would have very little impact on EC. Regarding EC, it is possible that LBM and total body water gains early into an exercise program can mask healthy BC changes. However, it is important to note that the values of BC that we used in our calculations were obtained at baseline and after the completion of the study, 3 months later. It is reasonable to assume that shifts in body water, would have had time to re-equilibrate over this time period. Additionally, two participants mostly trained on an ergometer, which could have reduced their EE across the intervention. The absence of change for the fitness level (VO_2peak_) across the intervention might suggest that the intensity was not high enough to cause an increase in oxygen consumption. Nevertheless, exercise intensities between 40 and 59% is normally recommended for sedentary individuals (2006). As well, the ramp type protocol used to prescribe the training program does not provide the steady state data necessary for accurately predict the HR/VO2 relationship. However, as mentioned earlier, the subject burden in this study was already very high. This would have entailed to add 3 additional exercise tests during the study, which was not feasible from a logistical standpoint. In addition, we used the same approach in both groups, so we can assume that the inflation of the caloric cost of exercise was the same for both groups. Finally, even though data were collected with the most available objective measures (i.e., DLW, DXA and accelerometry) and included both sides of the energy balance, the limitations associated with the non-continuous measures of EI and EE should not be underestimated, as previously discussed.

Data suggested a total EC of 96 % and a higher compensation in women training at MOD when compared to women training at LOW. This study adds new perspectives by suggesting that NSPA should be taken into consideration when investigating changes in body energy stores in response to exercise interventions. Finally, our study highlighted the limitations associated with the use of repeated acute measures to predict long-term outcomes, especially when the measurement of EI is involved.

## Author Contributions

M-ÈR and ÉD designed the research. M-ÈR, SJ-T, and J-CG conducted the research. M-ÈR, GF, JB, and ÉD conceptualized the analysis of the data. M-ÈR and GL analyzed the data. M-ÈR wrote the manuscript. SJ-T, GL, GF, JB, LD-S, J-CG, and ÉD critically appraised and approved the final version of the manuscript. ED had primary responsibility for final content.

## Conflict of Interest Statement

The authors declare that the research was conducted in the absence of any commercial or financial relationships that could be construed as a potential conflict of interest.

## Abbreviations

BC: body composition
BW: body weight
DLW: doubly labeled water
DXA: dual-energy X-ray absorptiometry
EC: energy compensation
EE: energy expenditure
EI: energy intake
FFM: fat free mass
FM: fat mass
HR: heart rate
LFPQ: leeds food preference questionnaire
LOW: low intensity
MOD: moderate intensity
NSPA: non-structured physical activity
TFEQ: three-factors eating questionnaire.
